# Evaluation of Shear Bond Strength of Total- and Self-etching Adhesive Systems after Application of Chlorhexidine to Dentin Contaminated with a Hemostatic Agent

**Published:** 2015-09

**Authors:** Farahnaz Sharafeddin, Hajar Farhadpour

**Affiliations:** 1 Biomaterial Research Center, Dept. of Operative Dentistry, School of Dentistry, Shiraz University of Medical Sciences, Shiraz, Iran.; 2 Postgraduate Student, Student Research Committee, Dept. of Operative Dentistry, School of Dentistry, Shiraz University of Medical Sciences, Shiraz, Iran.

**Keywords:** Chlorhexidine, Hemostatic agent, Self-etching adhesive, Shear, Total-etching adhesive

## Abstract

**Statement of the Problem:**

Hemostatic agents may influence the bond strength of different bonding agents. Also, chlorhexidine has shown positive effects on bond strength values and their combination effect has not been reported yet.

**Purpose:**

The aim of this study was to evaluate the effect of contamination with a hemostatic agent on shear bond strength (SBS) of total- and self-etching adhesive systems and the effect of chlorhexidine application after removal of the hemostatic agent.

**Materials and Method:**

In this experimental study, the occlusal enamel of each sixty caries-free mandibular molars was removed and their midcoronal dentin was exposed. The specimens were then mounted in auto-polymerizing resin 1mm apical to CEJ. Then, the specimens were divided into 6 groups (n=10) based on contamination with a hemostatic agent (H), application of chlorhexidine (CHX) and the adhesive system used; and then were classified as Group 1: Adper Single Bond (ASB); Group 2: H+ASB; Group 3: H+0.2% CHX+ASB; Group 4: Clearfil SE Bond (CSB); Group 5: H+CSB; Group 6: H+0.2% CHX+CSB. Then, composite resin rods (4×2 mm) were built up on the dentin surfaces and after thermocycling, the SBS (MPa) was evaluated. Statistical analysis was performed using two-way ANOVA and post hoc Tukey tests (*p*< 0.05).

**Results:**

There were statistically significant differences between bond strength values of group 1 (ASB) and group 2 (H+ASB) (*p*< 0.001) and group 1 (ASB) and group 3 (H+CHX+ASB) (*p*< 0.001). Similarly, significant differences were seen between group 4 (CSB) and group 5 (H+CSB) (*p*< 0.001) and between group 4 (CSB) and group 6 (H+CHX+CSB) (*p*< 0.001).

**Conclusion:**

Contamination with hemostatic agent reduced the SBS of both total- and self-etching adhesive systems. In addition, application of chlorhexidine after the removal of hemostatic agent had a negative effect on SBS of total- and self-etching adhesive systems.

## Introduction

Good adhesion to tooth structure is one of the prime prerequisites for tooth-colored restorations. Moisture control is essential in order to achieve a durable bond.[1-2] However, isolation against contaminants like saliva, sulcular fluid, blood, and handpiece lubricants, especially near the gingival margins or proximal boxes is difficult.[3]

Contamination with saliva and blood can reduce the dentin bond strength of total- and self-etching adhesive systems.[4-7] A thin film formed by blood proteins and macromolecules such as fibrinogen and platelets impairs adhesion to dentin. Long-term contamination with saliva also causes the formation of a mucin protein layer that impairs the bond.[8-9] Larger particles can be eliminated by rinsing but a reaction between the exposed collagen fibrils and the blood components may inhibit infiltration of primer into dentin.[8] In clinical situations that we have blood contamination, we can use hemostatic agents which are substances that precipitate proteins and are applied to hemorrhagic gingival tissues before placing restorations.[10-11] There are several kinds of hemostatic agents, such as aluminum chloride with a concentration of 5-25%. Having minimal systemic effects, this hemostatic agent is one of the most frequently used astringents that can precipitate proteins, constrict blood vessels, and extract fluid from tissues.[10] Hemostatic agents are highly acidic and can remove the smear layer and cause some degree of demineralization and their long-term application can even remove peritubular dentin.[11]

The effect of hemostatic agents on bond strength has been evaluated in some studies with conflicting results. Mostly, they have shown negative effects on bond strength values.[12-13] On the other hand, it has been shown in some studies that chlorhexidine has a positive effect on shear bond strength (SBS) of total- and self-etching adhesive systems.[14-15]

Generally, bond degradation occurs because of resin elution and alteration of collagen fibrils. Exposed collagen fibrils in the hybrid bond layer, not completely infiltrated by resin, are susceptible to degradation that is promoted by matrix metalloproteinase (MMP) enzymes in the saliva and dentin.[16-18] MMPs are a host cell-derived proteolytic enzyme family with a major role in tissue-destructive inflammatory diseases; they are responsible for degrading most of the extracellular matrix components including different types of collagen.[19-20]

Auto-degradation of collagen matrices in incompletely resin-infiltrated dentin can be prevented or minimized with the use of chlorhexidine.[14, 21] Its application has led to improved dentin microtensile bond strength after thermocycling,[15] decreasing loss of microtensile bond strength after 2 and 6 years of artificial aging in saliva.[22-23] Functional enzyme analysis of dentin powder also revealed low levels of collagenolytic activity after the use of chlorhexidine.[24]

Considering the negative effects of hemostatic agents on bond strength and the positive effects of chlorhexidine, the aim of this study was to evaluate the effect of contamination with a hemostatic agent and application of chlorhexidine after removal of the hemostatic agent on SBS of total- and self-etching adhesive systems. The null hypothesis tested was that contamination with hemostatic agent and application of chlorhexidine after removal of hemostatic agent would not affect the SBS of total- and self-etching adhesive systems. 

## Materials and Method

In this experimental study, sixty mandibular third molars without any caries, restorations or cracks were collected and used within one month after extraction. Then, the specimens were stored in 0.5% chloramine-T (Fisher Chemical; Fair Lawn, NJ, USA) solution for 24 hours. They were cleaned using an ultrasonic cleaner and used within one month after extraction. The occlusal enamel was removed using a high-speed diamond saw (Leitz 1600; Wetzlar, Germany) under a water coolant and the midcoronal dentin was exposed. Then the teeth were mounted in an auto-polymerizing acrylic resin block (2×2×1 cm) 1mm apical to CEJ and flat dentin surfaces were exposed. The exposed dentin surfaces were further polished with a 600-grit silicon carbide paper for 30 seconds under water irrigation to standardize the smear layer. The teeth were rinsed with distilled water to remove any debris. Then the specimens were randomly divided into 6 groups (n=10) based on contamination with hemostatic agent (Hemostop; Dentsply, Argentina), application of chlorhexidine (Sigma Aldrich, USA), and the adhesive agent chemical composition of aluminum chloride.

In group 1, etching was done with 37% phosphoric acid gel for 15 seconds. After rinsing and air-drying, and Adper Single Bond (ASB) (3M ESPE, USA) was applied as recommended by the manufacturer (Table 1).

In group 2, One drop of hemostatic agent solution (H) was applied directly to the dentin surface for 2 minutes, rinsed for 30 seconds and air-dried. Then the dentin surface was etched with 37% phosphoric acid gel for 15 seconds. After rinsing and air-drying, ASB was applied (H+ASB).

In group 3, One drop of the H was applied directly to the dentin surface for 2 minutes, rinsed for 30 seconds and air-dried. After that, 0.2% chlorhexidine (CHX) solution was applied with a microbrush. The next steps were similar to those in group 1 (H+CHX+ASB).

In group 4, Clearfil SE Bond (CSB) (Kuraray, Japan) was applied to exposed dentin surfaces as recommended by the manufacturer (Table 1).

**Table 1:** Adhesive systems, composition, and application mode

**Adhesive Systems **	**Composition **	**Application mode**
Adper Single Bond (3M, ESPE, St Paul, MN,USA)	Scotchbond etchant: 37% phosphoric acid Adhesive: Bis-GMA, HEMA, dimethacrylates, polyalkenoic acid copolymer, initiators, water and ethanol	1.Apply acid etch for 15s 2.Rinse for 15s. 3.Blot dry for 30s. 4.Apply one coat of adhesive for 10s. 5.Airdry for 10s at 20cm. 6.Light cure.
Clearfil SE Bond (Kuraray, Okayama, Japan)	Primer:MDP, HEMA, dimethacrylate Monomer, water, catalyst Bond: MDP, HEMA, dimethacrylate Monomer, microfiller, catalyst	1.Apply primer and leave for 20s. 2.Dry thorouly with mild air flow. 3.Apply bond. 4.Gentle air flow. 5.Light cure.

In group 5, One drop of H was applied directly to the dentin surface for 2 minutes, rinsed for 30 seconds and air-dried. Then, CSB was applied (H+CSB).

In group 6, One drop of H was applied directly to the dentin surface for 2 minutes, rinsed for 30 seconds and air-dried. After that, 0.2% CHX solution was applied with a microbrush. Finally, CSB was applied (H+CHX+CSB).

In all the groups, after application of the adhesive and light-curing for 20 seconds with an LED unit (Demetron; Kerr, USA) (1600 mW/cm²), a composite resin (Filtek Z250;3M ESPE, St. Paul, MN, USA) rod with A2 shade was built up on the dentin surface of each tooth using a split Teflon mold with an internal diameter of 4 mm and a height of 2 mm. Excess composite resin was removed with an explorer and then light-cured with an LED light-curing unit for 20 seconds. Then the specimens were stored in distilled water at 37°C in a water bath (Teledyne Hanau; Buffalo, N.Y, USA) for 24 hours. To simulate conditions of the oral cavity, the samples were thermocycled (Rika-Kogyo; Hachioji, Japan) for 1000 cycles between 5°C and 55°C with a 30-second dwell time and a 30-second transfer time.

Subsequently, the specimens were tested in shear mode using a chisel-shaped rod of a universal testing machine (Zwick; Roell, Z020, Germany) at a crosshead speed of 1 mm/min. The force at failure was recorded in Newtons (N); then, shear bond strength values were calculated in MPa. Statistical analysis was carried out with two-way ANOVA and post hoc Tukey tests (*p*< 0.05).

## Results

**Figure 1:**
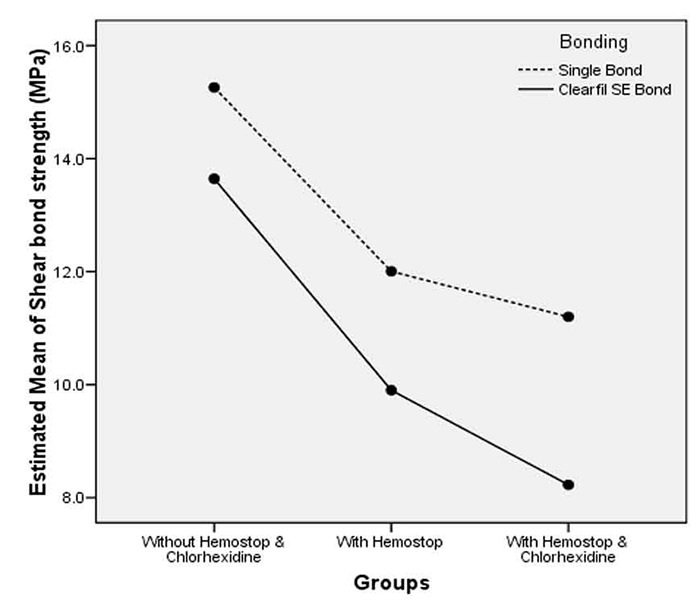
Estimated marginal means of shear bond strength values of two adhesive systems to dentin

The means and standard deviations of shear bond strength values are summarized in Table 2. Two-way ANOVA was used to evaluate the interactive effect of adhesive agents and materials (Hemostop and CHX). Regardless of the material (Hemostop and CHX) used, ASB showed significantly higher SBS values than CSB (*p*< 0.001).When the bonding effect was not taken into account, the control groups (groups 1 and 4) showed significantly higher SBS values than groups in which Hemostop and/or CHX were used (groups 2, 3, 5, and 6) (*p*< 0.001). There were no significant interactions between adhesive agents and materials such as Hemostop and CHX (*p*= 0.491). Therefore, the patterns of changes in mean values of SBS were similar in both adhesive systems (Figure 1).

**Table 2:** Mean and standard deviation of shear bond strength values of all groups (n=10)

**Groups**	**Mean (MPa)**	**Std.Deviation**
1(Single Bond)	15.26^a^	1.76
2(Hemostop+ Single Bond)	12^b^	2.03
3(Hemostop+chlorhexidine+ Single Bond)	11.2^b^	1.52
4(Clearfil SE Bond)	13.64^A^	1.49
5(Hemostop+ Clearfil SE Bond	9.9^B^	2.18
6(Hemostop+chlorhexidine+ Clearfil SE Bond)	8.22^B^	1.75

For pair-wise comparisons between the materials, post hoc Tukey tests were used. There were statistically significant differences between SBS values of group 1.

(ASB) and group 2 (H+ASB) (*p*< 0.001) and groups 1 (ASB) and 3 (H+CHX+ASB) (*p*< 0.001). Similarly, significant differences were seen between group 4 (CSB) and group 5 (H+CSB) (*p*< 0.001) and between groups 4 (CSB) and 6 (H+CHX+CSB) (*p*< 0.001). However, groups 2 (H+ASB) and 3 (H+CHX+ASB) showed no significant differences (*p*= 0.087). Likewise, no significant differences were seen between groups 5 (H+ CSB) and 6 (H+CHX+ CSB) (*p*= 0.087) (Table 2, 3). The Mean values followed by superscript with different letters indicate significant statistical differences at *p*< 0.05. The differences between three first groups are shown in different small letters. The differences between three second groups are shown in different capital letters.

**Table 3:** The Significancy of shear bond strength values of two adhesive systems to dentin

**Groups**	**Hemostop**	**Hemostop+Chlorhexidine**
Single Bond without Hemostop & Chlorhexidine	*p*= 0.0001	*p*=0.0001
Clearfil SE Bond without Hemostop & Chlorhexidine	*p*= 0.0001	*p*=0.0001

## Discussion

Hemostatic agents mostly have acidic properties (pH=0.7-3) and hydrophilic characteristics that may affect each stage of the bonding procedure. They cause some changes in dentin surfaces that could affect the hybrid layer quality after the use of total- and self- etching adhesive systems.[11] Thus, penetration of adhesives into dentin surface is prevented and ultimately affects SBS.[3]

The first part of this study evaluated the effect of contamination with a hemostatic agent on SBS of both total- and self-etching adhesive systems to dentin. The first part of our null hypothesis indicating that contamination with a hemostatic agent would not affect SBS of total- and self-etching adhesive systems was rejected. Thereafter; it was revealed that contamination with a hemostatic agent reduced the SBS of both total- and self-etching adhesive systems. Our findings were in line with those of a previous study by Arslan *et al.* who reported a decrease in SBS values after the application of hemostatic agent with both total- and self-etching adhesives.[12]

Many researches have reported hemostatic agent had a negative effect on SBS of self-etching adhesives. [11, 13, 25] This finding can be due to the weak acidity of Clearfil SE primer that can reduce its demineralizing effect on the dentin contaminated with hemostatic agent. Regarding the total-etching adhesive systems, different results have been obtained in different studies.[25, 27]

Kuphasuk *et al.* reported no significant effect of hemostatic agents on shear bond strength of total-etching adhesive systems,[25] which is different from our findings. Such dissimilarity might be attributed to different bonding agents used in our study. They used Excite adhesive agent but we used ASB, which is less acidic than Excite and might have led to a lower demineralizing effect on dentin and thus, a lower shear bond strength value.

A study by Ayo-Yusuf *et al.*[26] concluded that hemostatic agents can dissolve the smear layer because of their acidic properties and also can obturate the dentinal tubules and demineralize dentinal surface. Granular precipitates, which remain even after acid-etching, were noted on surfaces exposed to aluminum chloride hemostatic agent. The exposure of prepared dentin surface to hemostatic agents altered its morphology and reduced the dentin's susceptibility to acid-etching.[25-27]

The effect of chlorhexidine application after the removal of hemostatic agent was evaluated in the second part of this study and revealed that use of chlorhexidine after removal of the hemostatic agent reduced the SBS values and the second part of the null hypothesis was rejected. Application of chlorhexidine to the cavity prior to its restoration has been recommended in some studies.[27-30] Residual bacteria have been shown to proliferate from the smear layer even when there is good seal from the oral cavity[27] and this problem may be magnified by microleakage of composite restoration at the margins not ending on enamel.[28] A possible solution to this problem is treating the cavity preparation with chlorhexidine which has a broad spectrum of action; generally Gram-positive bacteria are more susceptible than Gram-negative ones, particularly *Streptococcus mutans* that seems to be more sensitive to chlorhexidine.[29] Currently, chlorhexidine is not considered only as an anti-microbial agent, but a potential adjuvant to establishing a better bond to dentin.[30] However, further studies are required in this repute since there is not a standard consensus and some studies have reported different results after the use of chlorhexidine.[30-31]

Regarding the step in which chlorhexidine is applied, some authors have used chlorhexidine after etching and showed higher bond strength values.[17, 21, 32] The rationale for this technique is that chlorhexidine has been shown to inhibit enzyme activities directly, and strongly inhibiting the inherent collagenolytic activity of mineralized dentin and thus, improving the bond of composite resin to dentin. Some other authors have used chlorhexidine before etching with the purpose of cleansing and disinfecting the dentin surface and showed no significant reduction in bond strength values.[31-32] Also, some investigators have reported no significant difference between chlorhexidine application, before or after etching of dentin.[34-35] In our study, we used chlorhexidine after removing the hemostatic agent and before etching in order to remove the microorganisms to reduce the probability of recurrent caries and restoration failure and also remnants of hemostatic agent that could interfere with the bond of composite to dentin.

In some previous studies the reported results were similar to our study. Ercan *et al.* reported that chlorhexidine solution had a negative effect on SBS of a self-etching adhesive system.[31] In a study by Herenio *et al.* the authors concluded that application of 2% chlorhexidine was deleterious for shear bond strength of self-etching adhesive to dentin after 24 hours of storage.[30]Although the concentration of chlorhexidine was different from that in our study, the results were the same. Chlorhexidine was incapable of stopping the bond degradation which routinely occurs by matrixmetalloproteinase enzymes at the dentin‒adhesive system interface.[36] The solution of 2% chlorhexidine before or after etching of the dentin did not interfere with microtensile bond strength of composite resin to dentin treated with Single Bond or Clearfil SE Bond bonding systems.[34]In another study, chlorhexidine instigated no significant effect on microtensile bond strength.[37]

Hebling *et al.* concluded that self-destruction of collagen may be arrested with the use of chlorhexidine.[14] Chang and Shin concluded that after thermocycling, chlorhexidine-treated groups exhibited better microtensile bond strength values.[15] Breschi *et al.* reported that chlorhexidine concentration (0.2% vs. 2%) was not important and both improved the microtensile bond strength values.[32] Similarly, better microtensile bond strength values were shown in chlorhexidine-treated groups in other studies.[22-23] In the present study, contrary to these studies, shear bond strength test was carried out and the results were different from microtensile tests. In tensile bond strength tests, a perpendicular alignment of the bonded interface to the loading axis is very important. Otherwise, bending stresses will develop. Specimen preparation is thus more technique sensitive for tensile bond strength tests than shear bond tests.[38] Hence, the current study desired to use shear test which is more routine.

In order to simulate conditions of the oral cavity, instead of storage, the specimens were subjected to a thermocycling procedure in the present study. This can lead to more precise and practical results. 

Loguercio *et al.* reported that stable bonds were maintained for up to 6 months under all the chlorhexidine conditions tested, irrespective of its concentration and application time and use of 0.2% chlorhexidine seemed to be sufficient to preserve the shear bond strength of total-etching adhesive to dentin.[17] Chlorhexidine was shown to prevent bond degradation,[19] which is not consistent with the results of the present study. This can probably be explained by the interactions that may occur between remnants of hemostatic agent, chlorhexidine, and the adhesive components in the present study. Any remaining particles from hemostatic agents could interfere with the chemical bond and micromechanical retention of adhesives, resulting in lower bond strengths.[39] Chlorhexidine may have diluted the adhesive components, resulting in a weaker bond. Chlorhexidine remnants on tooth substrate could influence bond strength test results due to moisture present on the dentin surface.[11] Another possible explanation is the effect of interaction between hemostatic agent and chlorhexidine that might have reduced the strengthening effect of chlorhexidine on bond strength. Further *in vitro* and *in vivo* studies are necessary to improve the understanding of the effect of interactions between different hemostatic agents and different concentrations of chlorhexidine and different bonding systems.

## Conclusion

Considering the limitations of this *in vitro* study, contamination with hemostatic agent reduced the shear bond strength of both total- and self-etching adhesive systems. Application of chlorhexidine after the removal of hemostatic agent did not improve the bond.
